# Real-time PCR expression profiling of genes encoding potential virulence factors in *Candida albicans *biofilms: identification of model-dependent and -independent gene expression

**DOI:** 10.1186/1471-2180-10-114

**Published:** 2010-04-16

**Authors:** Heleen Nailis, Soňa Kucharíková, Markéta Řičicová, Patrick Van Dijck, Dieter Deforce, Hans Nelis, Tom Coenye

**Affiliations:** 1 Laboratory for Pharmaceutical Microbiology, Universiteit Gent, Harelbekestraat 72, B-9000, Ghent, Belgium; 2 Department of Molecular Microbiology, VIB, Kasteelpark Arenberg 31 B-3001, Heverlee, Belgium; 3 Laboratory of Molecular Cell Biology, Institute of Botany and Microbiology, K.U. Leuven, Kasteelpark Arenberg 31, B-3001, Heverlee, Belgium; 4 Comenius University in Bratislava, Faculty of Natural Sciences, Department of Microbiology and Virology, Mlynska dolina B-2, 842 15 Bratislava, Slovakia; 5 Laboratory for Pharmaceutical Biotechnology, Universiteit Gent, Harelbekestraat 72, B-9000, Ghent, Belgium

## Abstract

**Background:**

*Candida albicans *infections are often associated with biofilm formation. Previous work demonstrated that the expression of *HWP1 *(hyphal wall protein) and of genes belonging to the *ALS *(agglutinin-like sequence), *SAP *(secreted aspartyl protease), *PLB *(phospholipase B) and *LIP *(lipase) gene families is associated with biofilm growth on mucosal surfaces. We investigated using real-time PCR whether genes encoding potential virulence factors are also highly expressed in biofilms associated with abiotic surfaces. For this, *C. albicans *biofilms were grown on silicone in microtiter plates (MTP) or in the Centres for Disease Control (CDC) reactor, on polyurethane in an in vivo subcutaneous catheter rat (SCR) model, and on mucosal surfaces in the reconstituted human epithelium (RHE) model.

**Results:**

*HWP1 *and genes belonging to the *ALS*, *SAP*, *PLB *and *LIP *gene families were constitutively expressed in *C. albicans *biofilms. *ALS1-5 *were upregulated in all model systems, while *ALS9 *was mostly downregulated. *ALS6 *and *HWP1 *were overexpressed in all models except in the RHE and MTP, respectively. The expression levels of *SAP1 *were more pronounced in both in vitro models, while those of *SAP2*, *SAP4 *and *SAP6 *were higher in the in vivo model. Furthermore, *SAP5 *was highly upregulated in the in vivo and RHE models. For *SAP9 *and *SAP10 *similar gene expression levels were observed in all model systems. *PLB *genes were not considerably upregulated in biofilms, while *LIP1-3, LIP5-7 *and *LIP9*-*10 *were highly overexpressed in both in vitro models. Furthermore, an elevated lipase activity was detected in supernatans of biofilms grown in the MTP and RHE model.

**Conclusions:**

Our findings show that *HWP1 *and most of the genes belonging to the *ALS, SAP *and *LIP *gene families are upregulated in *C. albicans *biofilms. Comparison of the fold expression between the various model systems revealed similar expression levels for some genes, while for others model-dependent expression levels were observed. This suggests that data obtained in one biofilm model cannot be extrapolated to other model systems. Therefore, the need to use multiple model systems when studying the expression of genes encoding potential virulence factors in *C. albicans *biofilms is highlighted.

## Background

*Candida albicans *is a dimorphic fungus that is part of the commensal microbial flora in many healthy human individuals [1]. When the host immune defences are impaired or when the normal microbial flora is disturbed, the fungus can cause superficial as well as severe systemic infections [1]. The transition from commensalism to parasitism is associated with transcriptional changes, and genes encoding adhesins and genes encoding hydrolytic enzymes are often expressed in *C. albicans *during infection [2,3]. In addition, the formation of hyphae and phenotypic switching are also involved in virulence of the fungus [2]. Genes belonging to the *ALS *(agglutinin-like sequence) gene family [4] and *HWP1 *(hyphal wall protein) [5] encode cell-surface associated glycosylphosphatidylinositol (GPI) anchored glycoproteins that mediate adhesion of *C. albicans *to mucosal surfaces [6]. Hwp1 in particular is a substrate for mammalian transglutaminase, and this adhesin mediates stable attachment of hyphae to epithelial cells [5]. *C. albicans *also contains three gene families that encode hydrolytic enzymes, including the *SAP *(secreted aspartyl protease), *LIP *(lipase) and *PL *(phospholipase) gene families [7-9]. Aspartyl proteases, lipases and phospholipases are enzymes secreted by the fungus which may contribute to colonization and infection by degrading components of host cell membranes [10].

Recently, it has become more and more clear that *C. albicans *infections are often associated with the formation of biofilms [11-13]. *C. albicans *biofilms are comprised of yeast cells and filaments that are attached to biotic or abiotic surfaces and embedded in an extracellular matrix [14,15]. Various model systems have been developed to study *C. albicans *biofilm biology on mucosal [16] and on abiotic surfaces [17-20]. Previous work demonstrated that the reconstituted human epithelium (RHE) is a valuable model to study *C. albicans *biofilms [21]. Using this model system, it was shown that the expression of *HWP1 *and of genes belonging to the *ALS*, *SAP, LIP *and *PLB *gene families is associated with biofilm growth on mucosal surfaces [21-25]. The expression of *ALS *genes and *HWP1 *has also been investigated in biofilms associated with abiotic surfaces [26-28]. Using mutant strains, it was demonstrated that Als1p, Als2p, Als3p and Hwp1 are important for biofilm growth in vitro and in vivo [6,29-32] and that Als1p/Als3p and Hwp1 have complementary roles in biofilm formation [33]. The determination of gene expression levels is often used to identify candidate genes involved in *C. albicans *biofilm formation [21-28]. However, it is known that the expression of *ALS, SAP, LIP and PLB *genes can be influenced by other factors such as the growth medium, temperature and other environmental conditions [6-9]. As such it can be anticipated that the biofilm model system can have a considerable impact on the expression levels of these genes.

The goal of the present study was to investigate the expression of genes encoding adhesins and genes encoding extracellular hydrolases in *C. albicans *biofilms grown in different model systems. This study was conducted to identify model-dependent and -independent expression levels of genes encoding potential virulence factors. The expression of *HWP1 *and of genes belonging to the *ALS*, *SAP*, *LIP *and *PLB *gene families was quantified in biofilms grown on mucosal surfaces as well as in biofilms grown on abiotic surfaces in vitro and in vivo, using real-time PCR. For this, *C. albicans *biofilms were grown on silicone in microtiter plates (MTP) or in the Centres for Disease Control (CDC) reactor, on polyurethane in an in vivo subcutaneous catheter rat (SCR) model, and on mucosal surfaces in the RHE model.

## Results

### C. albicans biofilm formation in the various biofilm model systems

**Figure 1:**
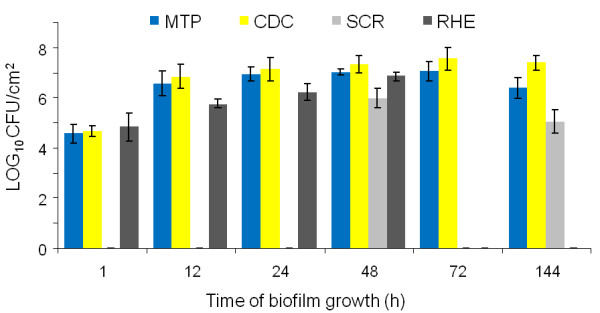
**Number of sessile *C. albicans *cells in biofilms grown in the various model systems**. Average number of culturable sessile cells (mean log_10 _CFU/cm^2 ^± SD) at selected time points during biofilm growth of *C. albicans *strain SC5314 in the various biofilm model systems. Biofilm growth was monitored on silicone in two in vitro models (MTP and CDC reactor), on polyurethane in an in vivo SCR model and on oral mucosal epithelium in the RHE model.

**Figure 2:**
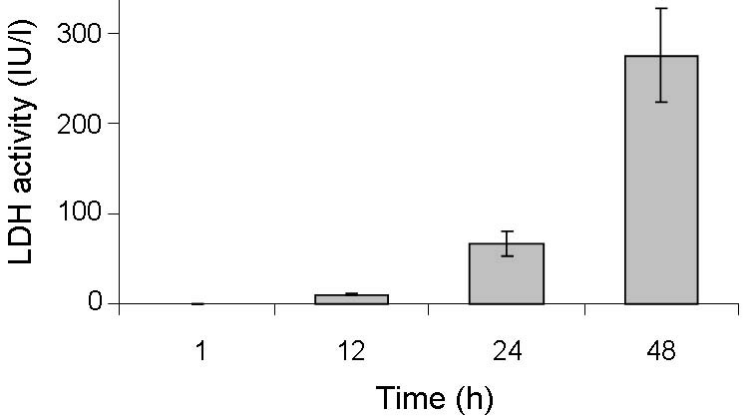
**LDH activity in the supernatant of sessile *C. albicans *cells**. LDH activity (IU/l at 37°C) at selected time points during biofilm growth of *C. albicans *strain SC5314 in the RHE model. Epithelial cell damage in the RHE model was correlated with release of the LDH marker.

The number of culturable sessile *C. albicans *cells was determined at selected time point during biofilm formation in the various model systems (Fig. 1). After 1 h of biofilm formation, the cell number was 4.6 ± 0.3 × 10^4 ^cells/cm^2 ^and 4.7 ± 0.2 × 10^4 ^cells/cm^2 ^in the MTP and in the CDC reactor, respectively. After 24 h, a mature biofilm was obtained in both in vitro models. Further incubation did not significantly increase the number of sessile cells. In the in vivo model, the cell number was 9.4 ± 0.4 × 10^5 ^cells/cm^2 ^after 48 h and 1.1 ± 0.5 × 10^5 ^cells/cm^2 ^after 144 h (Fig. 1). In the RHE model, the number of sessile cells was 6.9 ± 0.6 × 10^4 ^cells/cm^2 ^after 1 h, and the cell number gradually increased during further biofilm formation. After 48 h, 7.0 ± 0.2 × 10^7 ^cells/cm^2 ^were obtained in this model system (Fig. 1). No tissue damage was observed after 1 h in the RHE model (Fig. 2). The extracellular lactate dehydrogenase (LDH) activity released by damaged epithelial cells gradually increased, and severe tissue damage was observed after 48 h (Fig. 2).

### Percentage of filaments in biofilms

**Figure 3:**
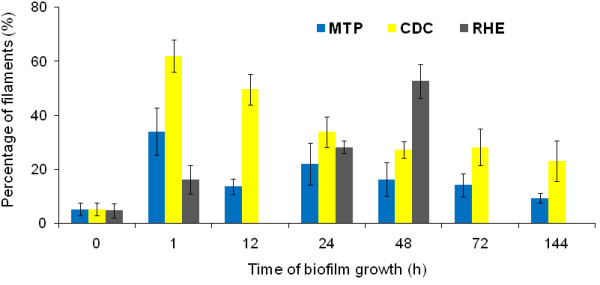
**Percentage of filaments in *C. albicans *biofilms**. Percentage (%) of filaments (with corresponding SD) at selected time points during biofilm growth of *C. albicans *strain SC5314 in the MTP, the CDC and the RHE model.

The percentage of filaments was determined in biofilms grown in the two in vitro models and in the RHE model, and results are shown in Fig. 3. The percentage of filaments in the start cultures (T = 0) were approximately 5%. In the CDC reactor, the percentage of filaments was 62 ± 6% (mean ± SD) after 1 h, and this percentage gradually decreased. After 144 h, only 23 ± 7% of all cells was filamentous. After 1 h of biofilm formation in the MTP, the percentage of filaments was approx. 2-fold lower than that observed in the CDC reactor (p < 0.05). The percentage of filaments also decreased during biofilm formation, and only 9 ± 2% of filaments was detected after 144 h of biofilm growth in the MTP. In the early stage of biofilm formation in the RHE model, the percentage of filaments is much lower compared to that in the two in vitro models (p < 0.05). After 1 h, only 16 ± 5.4% of filaments were detected in biofilms. However, the percentage of filaments gradually increased during biofilm formation in the RHE model, which is completely opposite to the results obtained in the two in vitro models. After 48 h, 53 ± 6.3% of all cells in biofilms were filamentous.

### Quality control of real-time PCR assays

Basic Local Alignment Search Tool (BLAST) analysis indicated that each primer pair was specific for a particular *C. albicans *gene, and would not cross-react with sequences from other organisms (data not shown). PCR efficiencies ranged between 90% and 110% for each of the primer pairs (data not shown), indicating that all real-time assays had similar good efficiencies. Gelelectrophoresis and melting curve analysis confirmed the presence of the expected PCR products only, and the absence of unwanted non-specific products (data not shown). Non-inoculated RHE failed to show evidence of gene expression (data not shown), confirming that each primer pair was specific for its corresponding *C. albicans *gene. Using the optimized real-time PCR assays, we found that *HWP1 *and all *ALS, SAP, LIP *and *PLB *genes were expressed at all time points during biofilm growth in all model systems tested (and also in the start cultures), as evidenced from a detectable C_t _value (C_t _< 35; data not shown).

### Expression levels of ALS genes and HWP1 in biofilms

The expression levels (expression in biofilms, relative to expression in start cultures) of *ALS *genes and *HWP1 *in biofilms at selected time points in the various model systems are shown in Additional file 1. *ALS1-5 *were overexpressed in biofilms grown in all model systems at several time points or during the entire time course. Furthermore, *HWP1 and ALS6 *were overexpressed in all model systems except in the MTP and RHE, respectively. *ALS9 *was only overexpressed in biofilms grown in the CDC reactor, but the fold upregulations were not particularly high. The fold expressions were model-dependent for most of the genes tested. Overexpression of *ALS3 *and *HWP1 *were more pronounced in biofilms grown in the in vivo model, while the expression levels of *ALS6 *were higher in the two in vitro models. Furthermore, the fold upregulations of *ALS4 *were more pronounced in biofilms grown in the in vivo and RHE models, while those of *ALS1, ALS2 *and *ALS5 *were higher in the two in vitro models and in the in vivo model.

### Expression levels of SAP genes in biofilms

The expression levels of *SAP *genes in biofilms at selected time points in the various model systems are shown in Additional file 2. All *SAP *genes (except *SAP3*) were upregulated in biofilms grown in all model systems at one or more time points. The expression levels of *SAP3 *were rather erratic, and this gene was not considerably upregulated in any of the model systems tested. For most of the *SAP *genes model-dependent expression levels were observed. In in vitro grown biofilms, *SAP1, SAP2, SAP4 *and *SAP6 *were highly upregulated, and the fold expression of *SAP2, SAP4 *and *SAP6 *were also high in the vivo model. Furthermore, *SAP5 *was highly upregulated in biofilms grown in the in vivo and RHE models. Only for *SAP9 *and *SAP10 *similar gene expression levels were observed in all model systems, although these genes were not expressed at a high level in biofilms.

### Expression levels of PLB genes in biofilms

The expression levels of *PLB *genes in biofilms at selected time points in the various model systems are given in Additional file 3. Overall, *PLB *genes were not considerably upregulated in biofilms, and only model-dependent differences in gene expression levels were observed. *PLB1 *was downregulated in biofilms grown in the MTP and in the in vivo and RHE models, but not in those grown in the CDC reactor. *PLB2 *was underexpressed in biofilms grown in the MTP and in the in vivo and RHE models (up to 12 h), but this gene was upregulated in biofilms grown in the CDC reactor and in the RHE model (after 24 h and 48 h).

### Expression levels of LIP genes in biofilms

The expression levels of *LIP *genes in biofilms at selected time points in the various model systems are shown in Additional file 3. *LIP2, LIP4 *and *LIP5 *were overexpressed in biofilms grown in all model systems at several time points or during the entire time course. Furthermore, *LIP1, LIP6, LIP9 *and *LIP10 *were upregulated in biofilms grown in the two in vitro models but not in the in vivo and RHE models. *LIP3 *was overexpressed in biofilms grown in the two in vitro models, while this gene was downregulated in the in vivo and RHE models. *LIP7 *was upregulated in biofilms grown in both in vitro models and in the in vivo model, but not in the RHE model. Similar results were obtained for *LIP8*, except that this gene was downregulated in biofilms grown in the MTP. For all the *LIP *genes (except *LIP4*), model-dependent gene expression levels were observed. *LIP1, LIP2, LIP9 *and *LIP10 *were highly overexpressed in biofilms grown in both in vitro models, whereas *LIP3 *and *LIP5-7 *were highly upregulated only in the CDC reactor. On the other hand, *LIP *genes were not expressed at a high level in biofilms grown in the in vivo and RHE models.

### Extracellular lipase activity

**Figure 4:**
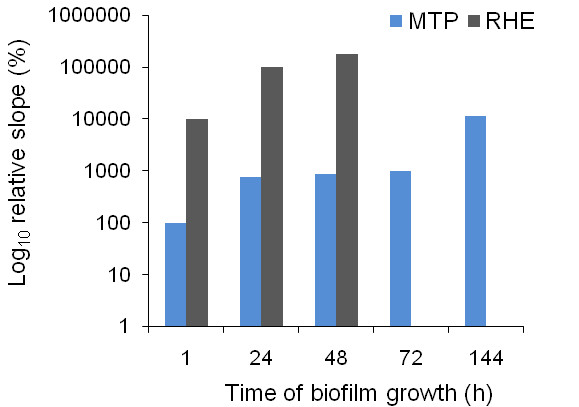
**Extracellular lipase activity of sessile *C. albicans *cells**. Extracellular lipase activity in the supernatant of sessile and planktonic *C. albicans *cells was determined using 4-MU palmitate. Relative slopes (%) of biofilms versus start cultures (derived from fluorescence-time curves) are shown for biofilms grown at selected time points in the MTP and RHE model.

Extracellular lipase activity in the supernatant derived from start cultures or from biofilms grown in the MTP and RHE model was determined using a fluorogenic substrate, 4-methylumbelliferyl (4-MU) palmitate. The relative slope (biofilms versus start cultures) of the fluorescence-time curves obtained from biofilms grown at selected time points in the MTP or RHE model is shown in Fig. 4. No differences in lipase activity were observed between biofilms grown for 1 h in the MTP and planktonic cells. Between 1 h and 24 h of biofilm growth in the MTP, lipase activity increased and then remained stable from 24 h up to 72 h. A marked increase in lipase activity was detected between 72 h and 144 h of biofilm growth in the MTP. In the RHE model after 1 h, lipase activity was approximately 100 fold higher than the lipase activity in planktonic cells. Lipase activity increased during further biofilm formation and was more than 1000 fold higher after 48 h of biofilm growth in the RHE model, compared to that in planktonic cells.

## Discussion

The aim of the present study was to investigate the expression of genes encoding adhesins and genes encoding extracellular hydrolases in *C. albicans *biofilms grown in different biofilm model systems. Biofilm formation on silicone progressed in a similar fashion in both in vitro model systems, although at later stages (72 h and 144 h), significantly lower cell numbers were obtained in the MTP than in the CDC reactor (p < 0.05). This is likely due to a continuous flow of fresh medium in the CDC reactor, absent in the MTP. In the in vivo model, cell numbers were significantly lower than in the two in vitro models (p < 0.05). Host factors and lack of direct accessibility to nutrients likely contribute to this phenomenon. In the RHE model, cell numbers were similar to those observed in the two in vitro models after 1 h. However, cell numbers increased more slowly during biofilm formation in the RHE model, which is likely due to the lack of direct accessibility to nutrients. In order to survive and grow, *C. albicans *needs to invade and destroy epithelial cells. Nevertheless, after 48 h cell numbers were similar to those observed in two in vitro models, indicating that a high-density biofilm was obtained. Green et al. previously showed that *C. albicans *inoculated on RHE forms a biofilm-like structure over the epithelial layer [21]. Furthermore, we observed no considerable tissue damage in the early stages of biofilm formation in the RHE model, whereas further biofilm growth led to a gradual increase in tissue destruction. Similar results were obtained in a previous study [25]. After 48 h, we found that the RHE tissue was almost completely degraded.

Using real-time PCR, the expression of *HWP1 *and of genes belonging to the *ALS, SAP, LIP *and *PLB *gene families was detected at all time points during biofilm growth in all model systems tested. It was previously shown that *ALS, HWP1, SAP *and *LIP *genes are expressed in the RHE model [21,22,24,25] and the expression of *PLB2 *but not *PLB1 *has also been detected in this model system [23]. However, the latter authors used reverse transcriptase PCR (RT-PCR) [23], whereas we used the more sensitive real-time PCR technique, and this probably explains why we were also able to detect *PLB1 *expression. The expression of *ALS1*, *ALS3 *and *HWP1 *has already been observed in biofilms associated with abiotic surfaces [26-28,31]. In the present study, we showed that not only *ALS1, ALS3 *and *HWP1*, but all the members of the *ALS, SAP*, *LIP *and *PLB *gene families were expressed in biofilms at all time points in all model systems tested. Together, we demonstrated that genes encoding adhesins and genes encoding extracellular hydrolases are constitutively expressed in biofilms grown on mucosal surfaces as well as in biofilms grown on abiotic surfaces in vitro and in vivo.

To identify model-dependent and -independent gene expression in *C. albicans *biofilms, the fold expression (expression level) of each gene was compared between the various model systems. Expression levels for each of the genes were determined by quantifying gene expression in biofilms relative to gene expression in the same reference condition (start cultures), using real-time PCR. By doing so, we found that *ALS1, ALS2 *and *ALS5 *were overexpressed in all model systems, but their fold upregulations were more pronounced in both in vitro models and in the in vivo model, compared to the RHE model. Using mutant strains, it was already demonstrated that Als1p and Als2p are involved in biofilm formation on abiotic surfaces [29,34]. Furthermore, *ALS4 *was highly upregulated in the two in vitro models, and was extremely overexpressed in the RHE and in vivo models. However, deletion of *ALS4 *did not significantly reduce biofilm formation on silicone and neither resulted in reduced biomass on RHE, but it is likely that Als2p compensates for the loss of *ALS4 *[34]. Our data clearly show high expression levels for *ALS4 *in biofilms grown on mucosal surfaces as well as on abiotic surfaces in vitro and in vivo, suggesting a role for Als4p in *C. albicans *biofilms. For *ALS6 *and *ALS9*, on the other hand, model-dependent up- and downregulations were observed. *ALS6 *was not overexpressed in the RHE model, which is not surprising as Als6p reduces adhesion of the fungus to buccal epithelial cells [35]. In both in vitro models and in the in vivo model, on the other hand, we observed an upregulation of *ALS6*. Using RT-PCR, it was previously shown that *ALS6 *was weakly expressed in biofilms grown on silicone [21]. However, using real-time PCR, we detected low C_t _values (i.e. high absolute mRNA levels) for *ALS6 *(data not shown). Furthermore, *ALS9 *is downregulated in the RHE model, in the MTP and in the vivo model, whereas this gene is slightly upregulated under flow conditions in the CDC reactor. It is possible that shear stress generated in the CDC reactor induces the expression of *ALS9*, although further research is needed to confirm this hypothesis. We also studied the expression of *ALS3 *and *HWP1*, two genes that encode hyphae-specific adhesins [36,37]. Their expression levels were higher in the CDC reactor than in the MTP, and the percentage of filaments was also higher in biofilms grown in the CDC reactor. Hyphae are known for their increased adhesive properties [13], and presumably shear stress in the CDC reactor triggers the fungus to form more filaments, which in turn express more *ALS3 *and *HWP1*. We also found that the percentage of filaments gradually decreased during biofilm formation in both in vitro models. It is known that contact-sensing induces filamentation in *C. albicans *[38], and therefore it is likely that initial contact of the fungus with the silicone results in filamentation. This could explain why young biofilms contain more filaments than mature ones in both in vitro models. Furthermore, *ALS3 *and *HWP1 *were highly upregulated in biofilms grown in the RHE model, and we found an increase in the percentage of filaments during biofilm formation in this model system. In order to grow in the RHE model, *C. albicans *needs to invade and destroy epithelial cells, and hyphae are known for their increased invasiveness [2,25]. *ALS3 *and *HWP1 *were also highly overexpressed in the in vivo model, which is not surprising as hyphae are the predominant form in biofilms grown in this model system [32].

Previous research demonstrated that members of the *SAP *gene family are expressed in biofilms associated with mucosal surfaces [24]. To investigate whether *SAP *genes are also highly expressed in biofilms associated with abiotic surfaces, the expression of each *SAP *gene was quantified in the various biofilm model systems. All *SAP *genes (except *SAP3*) were upregulated in the vitro and in vivomodels, supporting recent findings that sessile *C. albicans *cells associated with abiotic surfaces secrete more aspartyl proteases than planktonic cells [39]. In the RHE model, we also observed an overexpression of all *SAP *genes, except *SAP3*. When comparing the fold expression of *SAP *genes between the various model systems, we found that the expression levels of *SAP9 *and *SAP10 *were similar in all model systems, while for other *SAP *genes model-dependent expression levels were observed. The expression levels of *SAP1 *were more pronounced in both in vitro models, while those of *SAP2*, *SAP4 *and *SAP6 *were higher in the in vivo model. The expression levels of *SAP3 *were rather erratic in both in vitro models, and no considerable overexpression of this gene was found in the in vivo and RHE models. Furthermore, the expression levels of *SAP5 *were more pronounced in the in vivo model and also in the RHE model at later time points (from 12 h up to 48 h). In in vitro grown biofilms, *SAP1, SAP2, SAP4 *and *SAP6 *in particular are highly upregulated. It is known that the main function of Saps is to degrade proteins [9], but they were also found to play a role in cell-cell adhesion [40]. Hence, it is possible that Saps are important for adhesion and nutrient acquisition in in vitro grown biofilms, although this hypothesis requires further investigation. Furthermore, *SAP2*, *SAP4*, *SAP5 *and *SAP6 *were highly overexpressed in in vivo grown biofilms, while only *SAP5 *was highly upregulated in the RHE model. Recently, it was shown that *SAP5 *is the only gene that is upregulated as infection of the RHE progressed [24], and our findings are in agreement with this observation. Like Naglik et al. [24], we found no correlation between the expression of other *SAP *genes and LDH activity, indicating that only *SAP5 *may contribute to tissue damage in the RHE model. However, it was recently demonstrated that aspartyl proteases (including Sap5) are not required for invasion of the RHE [41], and this questions the role of Sap proteins in biofilms grown in the RHE model. It would be interesting to investigate whether the high expression of *SAP2, SAP4, SAP5 *and *SAP6 *in the in vivo model is associated with tissue damage of rats. On the other hand, the elevated expression of *SAP4-6 *in the in vivo model could also be associated with the presence of hyphae in biofilms. It is known that *SAP4-6 *are predominantly expressed in hyphae [9] and that hyphae are the predominant form in biofilms grown in the in vivo model [32]. For *SAP9 *and *SAP10*, similar gene expression levels were observed in all model systems. Although no considerable upregulations were seen for these genes, we detected much lower C_t _values for *SAP9 *(and to a lesser extent for *SAP10*) than for the other *SAP *genes (data not shown). In the RHE model, Naglik et al. [24] recently showed that *SAP9 *was the most highly expressed *SAP *gene. It is known that Sap9 and Sap10 are not secreted by the fungus, but are GPI anchored proteins that play a role in cell-surface integrity [42]. Based on our data, *SAP9 *(and to a lesser extent *SAP10*) are constitutively expressed at a high level in sessile cells, and it is possible that Sap9 and Sap10 play a cell surface-associated role in *C. albicans *biofilms.

For the *PLB *genes, only model-dependent differences in gene expression levels were observed. Overall, these genes were not considerably upregulated in *C. albicans *biofilms, and this is in agreement with a recent report in which it was shown that planktonic cells produce more phospholipases than biofilms [43]. We also found that *PLB *and *SAP *genes were simultaneously expressed in biofilms. It has previously been suggested that phospholipases and proteases have synergistic roles in tissue invasion in the RHE model [23]. Hence, phospholipases B could also contribute to tissue damage in the in vivo model. On the other hand, the role of phospholipases B in in vitro grown biofilms is more difficult to understand, but it is reasonable to propose that these enzymes play a role in nutrient acquisition. Based on our data, *PLB *genes are constitutively expressed in sessile cells in all model systems, although not at a high level, and further research is needed to reveal whether phospholipases B have important functions in *C. albicans *biofilms.

For most of the *LIP *genes, model-dependent gene expression levels were observed. However, the expression levels of *LIP *genes were rather similar in both in vitro models on the one hand, and in the in vivo and RHE models on the other hand. Based on our data, *LIP1, LIP2, LIP9 *and *LIP10 *were highly overexpressed in biofilms grown in both in vitro models, whereas *LIP3 *and *LIP5-7 *were highly upregulated only in the CDC reactor. On the other hand, *LIP *genes were not considerably upregulated in biofilms grown in the in vivo and RHE models. Although no high upregulations were seen in the latter model systems, all members of the *LIP *gene family were constitutively expressed in the in vivo and RHE models. We also investigated the extracellular lipase activity in the supernatant of sessile *C. albicans *cells in the MTP and RHE model. Lipase activity was significantly higher in biofilms grown in the RHE model, compared to that of biofilms grown in the MTP (p < 0.05). Furthermore, an increase in lipase activity during biofilm formation in the RHE model coincided with an increase in LDH activity of damaged epithelial cells. Hence, it could be proposed that lipases play a role in the invasion of epithelial tissue in the RHE model. On the other hand, the role of lipases in in vitro grown biofilms is not that obvious. It is possible that lipases play a role in nutrient acquisition [8], particularly in the MTP as nutrients become limited after prolonged biofilm growth. Together, our data demonstrate that *LIP *genes are upregulated in biofilms and extracellular lipases are produced by sessile *C. albicans *cells. However, the role and function of these secreted enzymes in *C. albicans *biofilms remains to be investigated.

Gene expression analysis is often used to identify candidate genes involved in *C. albicans *biofilm formation [21-28]. Previous studies have already examined the global transcriptional response in biofilms grown in particular model systems [26,44-46]. Similar to the in vitro models previously studied [26,31,45], the current study found an overexpression of *HWP1 *and of several genes belonging to the *ALS *gene family. In addition, analysis of gene expression in biofilms grown in the MTP and CDC also identified differences from previous studies. We found that most of the genes belonging to the *SAP *and *LIP *gene families are overexpressed in biofilms grown in vitro with or without flow. Recently, a global transcriptional analysis was performed in an vivo venous catheter biofilm model, and *ALS1, ALS2 *and *ALS4 *as well as *SAP5 *and *SAP10 *were upregulated in this model system [46]. In the present study we found an upregulation of *HWP1 *and of all *ALS *and *SAP *genes (except *ALS9*) in the in vivo subcutaneous catheter rat model. Similar to the venous catheter model [46], the current study observed an upregulation of several genes belonging to the *LIP *gene family and a downregulation of *PLB *genes. When comparing previously reported gene expression results from in vitro [26,44,45] or in vivo [46] biofilm experiments with the current data, both similarities and differences in gene expression were observed. This again highlights the fact that the biofilm model system can have a considerable impact on gene expression.

## Conclusions

In conclusion, we can state that *HWP1 *and most of the genes belonging to the *ALS, SAP *and *LIP *gene families are upregulated in *C. albicans *biofilms in all model systems tested. Future functional analyses of these genes in sessile *C. albicans *cells will allow us to better understand the exact roles of adhesins and extracellular hydrolytic enzymes in *C. albicans *biofilms. Comparison of the fold expression of genes encoding potential virulence factors between the two in vitro models, the in vivo model and the RHE model revealed similarities in expression levels for some genes, while for others model-dependent expression levels were observed. The present study indicates that gene expression data obtained from different biofilm model systems need to be carefully interpreted. We strongly believe that extrapolation of gene expression data from one model to another is not always feasible, and that it is recommended to use multiple biofilm model systems when studying gene expression in and/or testing anti-virulence strategies against *C. albicans *biofilms.

## Methods

### Strains

*C. albicans *strain SC5314 was used throughout the study. Cells were stored at -80°C in Microbank tubes (Prolab Diagnostics, Richmond Hill, ON, Canada) and routinely transferred to Sabouraud Dextrose Agar plates (SDA; Oxoid, Hampshire, UK). These were incubated at 37°C for 24 h.

### Biofilm growth in the MTP and CDC reactor

Start cultures were prepared by incubating *C. albicans *cells for 16 h in Sabouraud Dextrose Broth (SDB; Oxoid) at 37°C with shaking. Cells were subsequently washed three times with and finally resuspended in 1 ml 0.9% (w/v) NaCl. The biofilm inoculum was prepared by adding 0.4 ml of this suspension to 99.6 ml 1× Yeast Nitrogen Base (1× YNB; BD, Franklin Lakes, NJ, USA) supplemented with 50 mM glucose (Sigma, St. Louis, MO, USA) [28]. Silicone disks were prepared as described previously [20]. For the experiments in the MTP, silicone disks were placed into 24-well plates (TPP, Trasadingen, Switzerland) and one ml of the biofilm inoculum was added to each disk. Plates were incubated for 1 h at 37°C after which cells were washed three times with 1 ml 0.9% (w/v) NaCl. Disks were then transferred to new 24-well plates, 1 ml 1× YNB was added to each disk and plates were incubated at 37°C for up to 144 h. Biofilms were grown in the CDC reactor, as described previously [20], with some modifications. Undiluted medium (1× YNB) was used during the entire biofilm experiments and the medium was continuously pumped through the reactor starting from 1 h.

### Biofilm growth in the in vivo subcutaneous catheter rat model

In vivo biofilm growth was performed using an in vivo SCR model, as described previously [32]. Polyurethane triple lumen intravenous catheters were cut into segments of 1 cm (Arrow International, Reading, PA, USA) and treated overnight with bovine serum at 37°C. *C. albicans *cell suspensions were then added to the catheter segments and these were incubated for 90 min at 37°C. Catheters were then implanted under the skin of the back of specific pathogen-free Sprague Dawley rats, as described previously [32]. All animal experiments were carried out in agreement with European regulations regarding the protection and well-being of laboratory animals and were approved by the animal ethical committee of the Katholieke Universiteit Leuven (Leuven, Belgium). In each rat, 9 catheter segments were implanted and these were removed from the subcutaneous tissue after 48 h or 144 h, as described previously [32].

### Biofilm growth in the oral RHE model

The RHE model for oral candidiasis was used for ex vivo biofilm growth on oral human epithelial tissue. RHE tissue was obtained from SkinEthic Laboratories (Nice, France) and infection experiments were performed as described previously [16]. In brief, 0.5 cm^2 ^RHE surfaces were infected with 2 × 10^6 ^cells in 50 μl of PBS, and as a control 50 μl of PBS without *C. albicans *cells was used. The inoculated and non-inoculated RHE were incubated in maintenance medium (SkinEthic Laboratories) at 37°C with 5% CO_2 _at 100% humidity for up to 48 h.

### Lactate dehydrogenase assay

The RHE tissue damage caused by *C. albicans *was assessed by determining the LDH activity in the extracellular medium, as described previously [25]. The LDH activity was expressed in IU/l at 37°C and was determined from at least 4 independent experiments, with 2 replicates per experiment (n ≥ 8). Statistical significance of differences between the different time points of infection were determined by One-Way ANOVA using the SPSS 15.0 software (p < 0.05).

### Cell quantification

To enumerate the number of culturable sessile cells, plating was used. Silicone disks, RHE filters or polyurethane catheter segments were transferred to 10 ml 0.9% (w/v) NaCl, and sessile cells were removed from the surface by three cycles of 30 sec sonication (Branson 3510, 42 kHz, 100 W; Branson Ultrasonics Corporation, Danbury, CT, USA) and 30 sec vortex mixing. Using this procedure, all cells were removed from the surface and clumps of cells were broken apart, without affecting the viability of the cells (data not shown). Serial tenfold dilutions of the resulting cell suspensions were plated on SDA and plates were incubated for 24 h at 37°C, after which colonies were counted. The experiments were performed at least in triplicate with several replicates per experiment (n ≥ 12). The average number of sessile cells per cm^2 ^(with corresponding SD) was calculated. One-way ANOVA tests were carried out using SPSS 15.0 software to determine whether differences were statistically significant (p < 0.05).

### Solid phase cytometry

To determine the percentage of filaments in biofilms grown in the MTP, the CDC and the RHE model, a previously developed method based on solid phase cytometry was used [28]. Biofilms were grown and harvested as described above. Experiments were carried out in three-fold with several replicates per experiment (n ≥ 12), and the percentage of filaments (mean with corresponding SD) was determined. One-way ANOVA tests were carried out using SPSS 15.0 software to determine whether differences were statistically significant (p < 0.05).

### Lipase activity assay

Planktonic cells and biofilms grown in the MTP and RHE model were cultured as described above. Supernatant from biofilms and planktonic cells was collected and sterilized by filtration through 0.22 μm membranes (Millipore, Billerica, MA, USA). Extracellular lipase activity was determined using a fluorogenic substrate, 4- MU palmitate. 200 μl of sterile supernatant and 20 μl of the 4-MU ester (200 μg/ml in DMSO; Invitrogen, Carlsbad, CA, USA) were added to black 96-well plates (Perkin Elmer, Wellesley, MA, USA). Appropriate controls were included and plates were incubated at 37°C for 7 h. At regular time intervals, fluorescence was measured using a microtiter plate reader (Wallac Victor; Perkin Elmer). The excitation and emission wavelengths for 4-MU are 355 nm and 460 nm, respectively. Linear regression analysis was performed on the data and the relative slopes were calculated from the fluorescence-time curves of start cultures and biofilms as follows: (slope of the curve for biofilms/slope of the curve for start cultures)*100. Data were obtained in three independent experiments. One-way ANOVA tests were carried out using SPSS 15.0 software to determine whether differences were statistically significant (p < 0.05).

### Real-time PCR

Biofilms and start cultures were grown and harvested as described above. Samples were obtained from at least four independently-grown biofilms (n ≥ 4) and from six independently-grown start cultures (n = 6). RNA extraction and cDNA synthesis were performed as described previously [20]. Primers for the *ALS *genes were obtained from the study of Green et al. [47], and primers for the other genes and for the reference genes were designed using Primer Express software (Applied Biosystems, Foster City, CA, USA). Full-length gene sequences were obtained from the *C. albicans *database http://www.candidagenome.org/[48]. The specificity of each primer was checked by comparing its sequence to the *C. albicans *database using BLAST [49]. The sequences of the primers developed in the present study are given in Table 1 and Table 2, and for all the primers a concentration of 300 nM was used (except for *PMA1 *for which 600 nM was used). For *SAP7 *and *SAP8*, no good quality primers were obtained, and therefore these two genes were excluded from the present study. Real-time PCR was performed in 96-well plates using the ABI PRISM^® ^7000 apparatus (Applied Biosystems) and the MESA GREEN qPCR masterMix Plus for SYBR Assay I dTTP kit (Eurogentec, Seraing, Belgium). Five μl of 1:2 diluted cDNA samples and 20 μl of mastermix (containing the primers) were added to the plates. Real-time PCR reactions were performed at 95°C for 5 min, followed by 40 cycles of 15 s at 95°C and 1 min at 60°C. Following PCR, samples were subjected to incubations of increasing temperature starting from 60°C to 95°C to obtain melt curves. Samples were also subjected to gelelectrophoresis as described previously [20]. Control samples were included on each plate to ensure that multiple plates could be compared. Control reactions were also performed with RNA that had not been reverse transcribed in order to ensure that no genomic DNA was amplified during the PCR reactions. Real-time PCR data were normalized with the geometric mean of five reference genes. *ACT1*, *RIP*, *RPP2B*, *PMA1 *and *LSC2 *were used for this purpose, as they have previously shown to be stably expressed in *C. albicans *biofilms and planktonic cells [20]. Normalized data were then used to calculate the relative gene expression levels. An expression level corresponds to the expression of a gene in biofilms, grown at a particular time point in a particular model system, relative to its expression in start cultures (planktonic cells grown for 16 h in SDB). Mann-Whitney U tests were carried out using SPSS 15.0 software to determine whether differences in gene expression were statistically significant between biofilms and start cultures (p ≤ 0.05).

**Table 1:** Forward (FW) and reverse (RV) primers used in real-time PCR for the reference genes and for the *SAP *genes.

Gene	Orientation	Primer sequence (5' to 3')
*HWP1*	FW	GACCGTCTACCTGTGGGACAGT
	RV	GCTCAACTTATTGCTATCGCTTATTACA
*ACT1*	FW	TTTCATCTTCTGTATCAGAGGAACTTATTT
	RV	ATGGGATGAATCATCAAACAAGAG
*RPP2B*	FW	TGCTTACTTATTGTTAGTTCAAGGTGGTA
	RV	CAACACCAACGGATTCCAATAAA
*PMA1*	FW	TTGCTTATGATAATGCTCCATACGA
	RV	TACCCCACAACTTGGCAAGT
*RIP*	FW	TGTCACGGTTCCCATTATGATATTT
	RV	TGGAATTTCCAAGTTCAATGGA
*LSC2*	FW	CGTCAACATCTTTGGTGGTATTGT
	RV	TTGGTGGCAGCAATTAAACCT
*SAP1*	FW	AACCAATAGTGATGTCAGCAGCAT
	RV	ACAAGCCCTCCCAGTTACTTTAAA
*SAP2*	FW	GAATTAAGAATTAGTTTGGGTTCAGTTGA
	RV	CCACAAGAACATCGACATTATCAGT
*SAP3*	FW	CAGCTTCTGAATTTACTGCTCCATT
	RV	TCCAAAAAGAAGTTGACATTGATCA
*SAP4*	FW	AAACGGCATTTGAATCTGGAA
	RV	CAAAAACTTAGCGTTATTGTTGACACT
*SAP5*	FW	CCAGCATCTTCCCGCACTT
	RV	GCGTAAGAACCGTCACCATATTTAA
*SAP6*	FW	TGGTAGCTTCGTTGGTTTGGA
	RV	GCTAACGTTTGGTCTACTAGTGCTCATA
*SAP9*	FW	AAAGCAGCAGCGGCAGTACT
	RV	ATCCAAAACAACACCCGTGGTA
*SAP10*	FW	CCTTATTCGAACCGATCTCCAA
	RV	CAATGCCTCTTATCAACGACAAGA

**Table 2:** Forward (FW) and reverse (RV) primers used in real-time PCR for the *PLB *and *LIP *genes.

Gene	Orientation	Primer sequence (5' to 3')
*PLB1*	FW	GGTGGAGAAGATGGCCAAAA
	RV	AGCACTTACGTTACGATGCAACA
*PLB2*	FW	TGAACCTTTGGGCGACAACT
	RV	GCCGCGCTCGTTGTTAA
*LIP1*	FW	AGCCCAACCAGAAGCTAATGAA
	RV	TGATGCAAAAGTCGCCATGT
*LIP2*	FW	GGCCTGGATTGATGCAAGAT
	RV	TTGTGTGCAGACATCCTTGGA
*LIP3*	FW	TCTCACCGAGATTGTTGTTGGA
	RV	GTTGGCCATCAAATCTTGCA
*LIP4*	FW	GCGCTCCTGTTGCTTTGACT
	RV	ACACGGTTTGTTTTCCATTGAA
*LIP5*	FW	TGGTTCCAAAAATACCCGTGTT
	RV	CGACAATAGGGACGATTTGATCA
*LIP6*	FW	AAGAATCTTCCGACCTGACCAA
	RV	ATATGCACCTGTTGACGTTCAAA
*LIP7*	FW	AACTGATATTTGCCATGCATTAGAAA
	RV	CCATTCCCGGTAACTAGCATGT
*LIP8*	FW	CAACAATTGCTAAAATCGTTGAAGA
	RV	AGGGATTTTTGGCACTAATTGTTT
*LIP9*	FW	CGCAAGTTTGAAGTCAGGAAAA
	RV	CCCACATTACAACTTTGGCATCT
*LIP10*	FW	CACCTGGCTTAGCAGTTGCA
	RV	CCCAGCAAAGACTCATTTTATTCA

## Authors' contributions

HN participated in the design of the study, performed the experimental procedures, carried out the data analysis, and drafted the manuscript. SK and MR helped to perform the experimental procedures. PVD and DD helped in the design of the study and in the draft of the manuscript. HJN participated in the coordination of the study and helped to draft the manuscript. TC conceived the study and helped to draft the manuscript. All authors read and approved the final manuscript.

## Supplementary Material

Additional file 1 **Table S1**. Expression levels of *ALS *genes and *HWP1 *in biofilms grown in the various model systems. Click here for file

Additional file 2 **Table S2**. Expression levels of *SAP *genes in biofilms grown in the various model systems. Click here for file

Additional file 3 **Table S3**. Expression levels of *PLB *and *LIP *genes in biofilms grown in the various model systems. Click here for file
