# Supplementary material for: Real-time PCR expression profiling of genes encoding potential virulence factors in Candida albicans biofilms: identification of model-dependent and -independent gene expression
Source: BMC Microbiol. 2010 Apr 16;10:114. doi: 10.1186/1471-2180-10-114 (PMC2862034; doi:10.1186/1471-2180-10-114)
Supplement: Additional file 1 — Table S1. Expression levels of ALS genes and HWP1 in biofilms grown in the various model systems. [file 1471-2180-10-114-S1.PDF]

Table S1: **Expression levels of *ALS* genes and *HWP1* in biofilms grown in the various model systems**. Gene expression levels were defined as the expression of a gene in biofilms, grown at a particular time point in a particular model system, relative to its expression in start cultures. Normalization of gene expression data was performed using the geometric mean of five stably expressed reference genes [20]. All expression values shown were statistically significant ($p \leq 0.05$); when gene expression was not statistically significant between biofilms and start cultures ($p > 0.05$), the gene expression levels were replaced by NS.

Biofilms	*ALS1*	*ALS2*	*ALS3*	*ALS4*	*ALS5*	*ALS6*	*ALS9*	*HWP1*
**MTP-1h**	9.8 ± 1.8	2.9 ± 0.5	6.0 ± 2.3	8.8 ± 3.5	6.1 ± 1.6	4.9 ± 1.0	-2.4 ± 0.8	NS
**MTP-12h**	16.8 ± 2.0	2.4 ± 0.9	NS	23.4 ± 9.3	8.9 ± 2.3	2.8 ± 1.1	NS	NS
**MTP-24h**	13.9 ± 0.9	11.9 ± 4.7	NS	35.6 ± 11.6	26.6 ± 9.2	NS	NS	NS
**MTP-48h**	12.2 ± 1.7	4.6 ± 1.5	NS	31.8 ± 11.3	12.4 ± 3.1	3.7 ± 0.5	NS	-7.6 ± 5.1
**MTP-72h**	9.8 ± 1.5	34.1 ± 27.9	NS	41.4 ± 12.8	73.3 ± 34.1	28.6 ± 20.1	NS	NS
**MTP-144h**	NS	10.3 ± 2.8	NS	23.3 ± 8.0	16.2 ± 4.7	11.5 ± 4.3	NS	NS
**CDC-1h**	NS	2.6 ± 1.3	9.5 ± 5.6	9.6 ± 3.2	9.3 ± 4.2	NS	NS	15.5 ± 14.3
**CDC-12h**	10.1 ± 1.8	20.8 ± 20.4	2.9 ± 1.2	14.2 ± 7.8	36.1 ± 27.6	35.1 ± 24.7	3.4 ± 1.6	6.8 ± 5.7
**CDC-24h**	5.1 ± 0.8	15.9 ± 14.9	2.1 ± 0.7	12.9 ± 9.0	34.1 ± 23.1	16.7 ± 13.2	3.0 ± 1.2	5.5 ± 4.0
**CDC-48h**	3.8 ± 0.4	2.4 ± 0.7	2.1 ± 0.6	NS	4.7 ± 1.9	NS	2.1 ± 0.5	NS
**CDC-72h**	5.7 ± 1.9	40.0 ± 5.6	NS	11.8 ± 3.9	41.8 ± 11.0	32.9 ± 5.4	2.6 ± 0.6	NS
**CDC-144h**	4.1 ± 0.2	7.2 ± 2.2	NS	8.9 ± 4.1	33.8 ± 16.0	17.8 ± 10.1	2.1 ± 0.6	NS
**SCR-48h**	21.6 ± 6.6	69.1 ± 21.8	38.8 ± 11.4	144.1 ± 63.9	63.4 ± 31.8	3.2 ± 1.0	NS	318.9 ± 219.5
**SCR-144h**	16.9 ± 1.8	33.8 ± 5.8	9.7 ± 2.7	233.5 ± 107.9	26.6 ± 13.5	6.7 ± 4.9	-8.1 ± 6.0	84.5 ± 63.8
**RHE-1h**	6.1 ± 0.3	3.6 ± 0.5	9.0 ± 3.1	19.7 ± 5.7	4.0 ± 1.0	NS	-9.7 ± 2.1	21.4 ± 13.7
**RHE-12h**	8.1 ± 1.1	NS	NS	26.2 ± 7.7	4.8 ± 1.3	NS	-5.5 ± 1.2	24.7 ± 15.6
**RHE-24h**	6.2 ± 0.6	5.5 ± 1.3	9.1 ± 2.7	146.6 ± 57.1	6.9 ± 1.7	NS	-3.4 ± 0.7	32.3 ± 20.4
**RHE-48h**	4.9 ± 0.6	10.3 ± 2.2	NS	313.2 ± 101.4	8.1 ± 2.0	NS	-2.8 ± 0.6	8.1 ± 5.1
