# Supplementary material for: Real-time PCR expression profiling of genes encoding potential virulence factors in Candida albicans biofilms: identification of model-dependent and -independent gene expression
Source: BMC Microbiol. 2010 Apr 16;10:114. doi: 10.1186/1471-2180-10-114 (PMC2862034; doi:10.1186/1471-2180-10-114)
Supplement: Additional file 2 — Table S2. Expression levels of SAP genes in biofilms grown in the various model systems. [file 1471-2180-10-114-S2.PDF]

Table S2: **Expression levels of *SAP* genes in biofilms grown in the various model systems.** Gene expression levels were defined as the expression of a gene in biofilms, grown at a particular time point in a particular model system, relative to its expression in start cultures. Normalization of gene expression data was performed using the geometric mean of five stably expressed reference genes [20]. All expression values shown were statistically significant ($p \leq 0.05$); when gene expression was not statistically significant between biofilms and start cultures ($p > 0.05$), the gene expression levels were replaced by NS.

**Biofilms**	***SAP1***	***SAP2***	***SAP3***	***SAP4***	***SAP5***	***SAP6***	***SAP9***	***SAP10***
**MTP-1h**	2.9 ± 1.1	4.2 ± 1.3	NS	2.1 ± 0.7	2.8 ± 0.8	13.3 ± 3.6	2.5 ± 0.5	NS
**MTP-12h**	17.7 ± 6.4	3.7 ± 1.0	NS	NS	NS	5.4 ± 1.8	4.0 ± 0.9	NS
**MTP-24h**	28.1 ± 13.2	5.7 ± 2.2	NS	NS	NS	6.6 ± 6.2	NS	3.2 ± 0.9
**MTP-48h**	23.0 ± 8.3	2.5 ± 0.6	-6.4 ± 1.5	NS	NS	2.0 ± 0.4	NS	NS
**MTP-72h**	128.4 ± 69.5	15.3 ± 8.0	NS	NS	NS	29.2 ± 27.8	2.0 ± 0.4	6.8 ± 2.6
**MTP-144h**	19.5 ± 8.1	3.9 ± 1.2	NS	3.8 ± 1.8	NS	8.9 ± 3.0	NS	2.4 ± 0.7
**CDC-1h**	23.3 ± 12.4	NS	-2.6 ± 1.2	NS	NS	NS	NS	NS
**CDC-12h**	24.2 ± 15.4	9.7 ± 7.1	NS	17.9 ± 19.3	NS	26.5 ± 28.3	4.8 ± 0.9	6.6 ± 3.5
**CDC-24h**	9.0 ± 6.5	6.5 ± 4.6	NS	NS	4.6 ± 2.7	19.8 ± 18.3	2.4 ± 1.0	4.2 ± 2.0
**CDC-48h**	NS	2.9 ± 0.9	NS	NS	NS	NS	3.4 ± 0.6	4.5 ± 1.3
**CDC-72h**	10.4 ± 4.4	11.6 ± 3.4	5.4 ± 0.4	19.3 ± 6.7	5.0 ± 1.5	41.8 ± 9.6	2.2 ± 0.5	5.7 ± 1.6
**CDC-144h**	6.4 ± 2.6	12.4 ± 5.5	NS	15.9 ± 10.5	NS	21.9 ± 17.9	NS	NS
**SCR-48h**	4.4 ± 1.8	37.8 ± 25.1	NS	51.1 ± 26.3	158.1 ± 80.6	211.3 ± 93.5	5.6 ± 1.6	3.1 ± 1.1
**SCR-144h**	NS	10.6 ± 8.0	2.9 ± 0.4	49.2 ± 15.0	62.3 ± 22.8	176.2 ± 34.4	5.4 ± 1.3	NS
**RHE-1h**	NS	4.0 ± 1.0	NS	NS	6.0 ± 1.6	6.0 ± 1.9	NS	2.2 ± 0.5
**RHE-12h**	NS	3.6 ± 0.9	NS	NS	10.5 ± 2.8	NS	NS	NS
**RHE-24h**	2.3 ± 0.8	5.3 ± 1.5	NS	NS	42.9 ± 12.9	4.4 ± 0.9	2.6 ± 0.5	NS
**RHE-48h**	3.0 ± 1.1	5.8 ± 1.4	NS	2.6 ± 0.8	75.4 ± 19.5	7.5 ± 1.7	2.4 ± 0.4	NS
