# Supplementary material for: Real-time PCR expression profiling of genes encoding potential virulence factors in Candida albicans biofilms: identification of model-dependent and -independent gene expression
Source: BMC Microbiol. 2010 Apr 16;10:114. doi: 10.1186/1471-2180-10-114 (PMC2862034; doi:10.1186/1471-2180-10-114)
Supplement: Additional file 3 — Table S3. Expression levels of PLB and LIP genes in biofilms grown in the various model systems. [file 1471-2180-10-114-S3.PDF]

Table S3: **Expression levels of *PLB* and *LIP* genes in biofilms grown in the various model systems.** Gene expression levels were defined as the expression of a gene in biofilms, grown at a particular time point in a particular model system, relative to its expression in start cultures. Normalization of gene expression data was performed using the geometric mean of five stably expressed reference genes [20]. All expression values shown were statistically significant ($p \leq 0.05$); when gene expression was not statistically significant between biofilms and start cultures ($p > 0.05$), the gene expression levels were replaced by NS.

| Biofilms | *PLB1* | *PLB2* | *LIP1* | *LIP2* | *LIP3* | *LIP4* | *LIP5* | *LIP6* | *LIP7* | *LIP8* | *LIP9* | *LIP10* |
|---|---|---|---|---|---|---|---|---|---|---|---|---|
| **MTP-1h** | NS | NS | NS | 3.2 ± 1.0 | NS | NS | 2.6 ± 0.9 | NS | NS | NS | NS | NS |
| **MTP-12h** | NS | NS | NS | NS | NS | NS | NS | NS | NS | NS | NS | 3.0 ± 1.5 |
| **MTP-24h** | NS | NS | NS | NS | NS | NS | NS | NS | NS | NS | NS | NS |
| **MTP-48h** | -5.5 ± 2.4 | -1.5 ± 0.2 | NS | NS | NS | NS | NS | NS | NS | -4.2 ± 1.9 | NS | NS |
| **MTP-72h** | NS | NS | 10.7 ± 8.1 | 272.5 ± 80.1 | NS | NS | NS | NS | NS | NS | 19.0 ± 19.1 | 51.3 ± 59.9 |
| **MTP-144h** | NS | NS | 33.1 ± 18.2 | 10.6 ± 5.3 | 2.6 ± 0.9 | 2.5 ± 0.6 | 4.9 ± 2.2 | 6.6 ± 3.1 | 5.3 ± 2.0 | NS | 10.1 ± 2.7 | 16.0 ± 7.6 |
| **CDC-1h** | NS | NS | NS | NS | NS | NS | NS | NS | 1.5 ± 1..2 | NS | NS | NS |
| **CDC-12h** | NS | NS | 15.4 ± 15.2 | 23.7 ± 27.1 | 10.0 ± 10.9 | NS | NS | 12.1 ± 10.8 | 13.0 ± 12.4 | NS | 16.8 ± 15.0 | 47.3 ± 46.3 |
| **CDC-24h** | NS | NS | 11.2 ± 9.5 | NS | NS | NS | NS | 11.8 ± 9.0 | 28.7 ± 8.8 | 3.9 ± 2.1 | 15.9 ± 12.5 | 34.0 ± 35.8 |
| **CDC-48h** | NS | 2.2 ± 0.1 | NS | NS | NS | NS | NS | NS | -1.0 ± 0.2 | NS | NS | NS |
| **CDC-72h** | NS | NS | 20.3 ± 6.9 | 50.7 ± 12.7 | 16.1 ± 2.8 | 5.0 ± 1.0 | 23.7 ± 7.3 | 16.2 ± 6.5 | 21.6 ± 3.7 | 5.1 ± 2.1 | 24.7 ± 9.4 | 94.0 ± 11.5 |
| **CDC-144h** | NS | 3.0 ± 1.6 | 15.9 ± 9.6 | 28.8 ± 34.5 | NS | 3.7 ± 2.1 | NS | NS | 11.7 ± 8.8 | 7.6 ± 3.3 | 14.4 ± 8.8 | 39.9 ± 34.8 |
| **SCR-48h** | -4.8 ± 2.2 | NS | NS | 4.8 ± 1.7 | -2.5 ± 0.9 | 4.0 ± 1.1 | 5.7 ± 2.3 | NS | 2.1 ± 0.4 | 4.2 ± 1.9 | NS | NS |
| **SCR-144h** | -7.1 ± 2.2 | -1.4 ± 0.1 | NS | 4.7 ± 1.8 | NS | 2.5 ± 0.4 | NS | NS | 3.4 ± 2.5 | 2.5 ± 1.0 | NS | NS |
| **RHE-1h** | -7.3 ± 2.2 | -1.4 ± 0.1 | NS | 4.2 ± 1.6 | NS | NS | NS | NS | NS | NS | NS | NS |
| **RHE-12h** | -9.8 ± 3.3 | -1.4 ± 0.1 | NS | 2.8 ± 0.7 | NS | NS | NS | NS | NS | NS | NS | NS |
| **RHE-24h** | -3.0 ± 0.9 | 3.2 ± 0.9 | NS | 3.0 ± 0.7 | -3.2 ± 0.8 | NS | 2.6 ± 0.8 | NS | NS | NS | NS | NS |
| **RHE-48h** | -3.1 ± 2.8 | 2.0 ± 1.7 | NS | 2.8 ± 0.6 | -3.4 ± 0.4 | 2.0 ± 0.2 | 3.0 ± 0.9 | NS | NS | NS | NS | NS |
